# Supplementary material for: Early life gut microbiota sustains liver-resident natural killer cells maturation via the butyrate-IL-18 axis
Source: Nat Commun. 2023 Mar 27;14:1710. doi: 10.1038/s41467-023-37419-7 (PMC10043027; doi:10.1038/s41467-023-37419-7)
Supplement: Supplementary file 1 — Supplementary Information [file 41467_2023_37419_MOESM1_ESM.pdf]

Supplemental information

Early life gut microbiota sustains liver-resident natural killer cells maturation via butyrate-IL-18 axis

Panpan Tian[1], Wenwen Yang[1], Xiaowei Guo[1], Tixiao Wang[1], Siyu Tan[1], Renhui Sun[1], Rong Xiao[1], Yuzhen Wang[1], Deyan Jiao[1], Yachen Xu[1], Yanfei Wei[1], Zhuanchang Wu[1,2], Chunyang Li[3], Lifen Gao[1,2], Chunhong Ma [1,2*], Xiaohong Liang [1,2*]

This file includes:

Supplementary Figures 1 to 11

Supplementary Tables 1 to 2

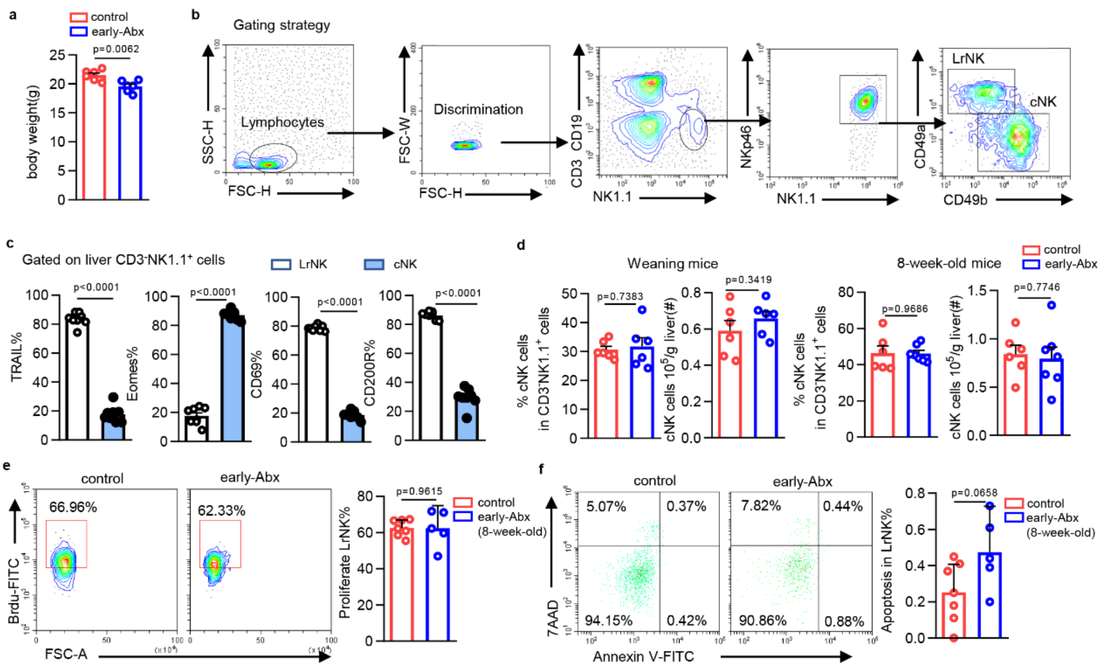

Supplementary Figure1 Effects of early-Abx exposure on liver NK cells.

(a) Body wight of control and early-Abx mice (n=6 per group). **(b)** Representative plot showing the gating strategy for liver CD3$^-$CD19$^-$NK1.1$^+$NKp46$^+$CD49a$^+$CD49b$^-$(LrNK) and CD3$^-$CD19$^-$NK1.1$^+$NKp46$^+$CD49a$^-$CD49b$^+$(cNK) cells. **(c)** Representative FACS plots and the percentages of TRAIL, Eomes, CD69 and CD200R of C57BL/6 mice (n=6 per group). **(d)** Bar graphs for the percentage and absolute number of cNK cells from control and early-Abx mice (weaning mice: n=6 per group; 8-week-old mice: control n=6, early-Abx n=7). **(e)** FCM analysis of Brdu level in LrNK cells from control and early-Abx mice (control n=7 and early-Abx n=5). **(f)** FACS plots and graphs show LrNK cell viability from control and early-Abx mice determined by Annexin V/7-AAD staining (control n=7 and early-Abx n=5). Each symbol represents data from an individual mouse, and error bars represent SEM per group in one experiment. Data were analyzed using two-tailed Student's t-test. **, P < 0.01; ****, P < 0.0001; ns, no significance. All experiments were repeated two or three independent experiments.

Source data are provided as a Source Data file.

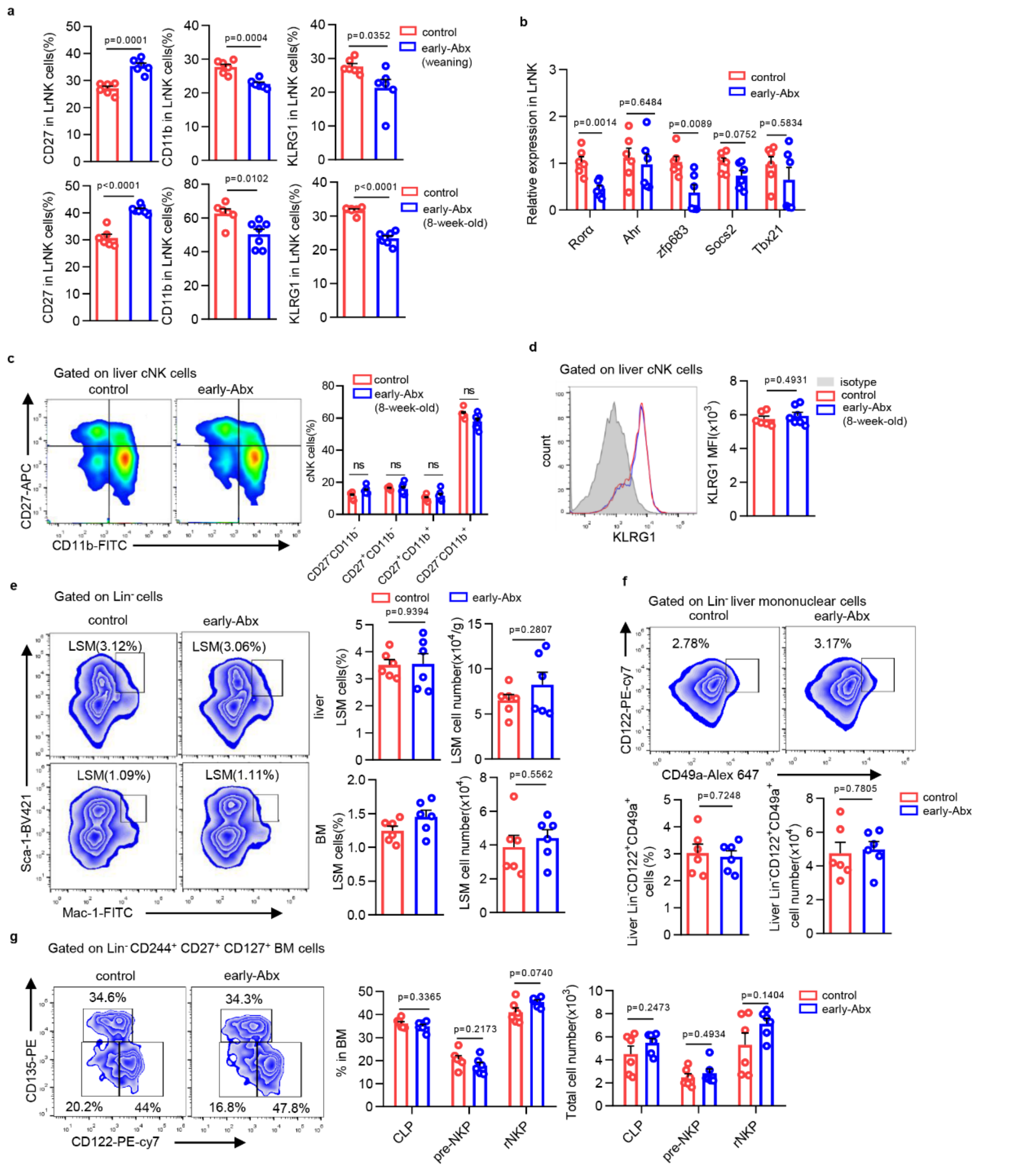

Supplementary Figure 2 The impact of early-Abx on NK cell development and maturation.

(a) Representative FACS plots and bar graph for the percentage of CD27, CD11b and KLRG1 in LrNK cell subsets from weaning and 8-week-old control or early-Abx mice (weaning, n=6 per group. 8week-old, control n=6, early-Abx n=7). **(b)** RT-qPCR analysis of indicated transcription factors expression level in purified LrNK cells from control or early-Abx mice (n=6 per group). **(c)** Representative FACS plots and bar

graph for the expression of CD11b/CD27 in cNK cells from control and early-Abx mice (control n=6 and early-Abx n=7). **(d)** Representative FACS plots and bar graph for the expression (MFI) of KLRG1 in cNK cell subsets from control and early-Abx mice (control n=6 and early-Abx n=7). **(e)** Representative FACS plots and bar graphs for the percentages and absolute number of LSM cells in bone marrow and liver from control and early-Abx mice (n=6 per group). **(f)** Representative FACS plots and bar graphs for the percentages and absolute number of Lin^{-} $CD122^{+}$ $CD49a^{+}$ progenitor cells in liver from control and early-Abx mice (n=6 per group). **(g)** Representative FACS plots and bar graphs for the percentages and absolute number of CLP, pre-NKP and rNKP cells in bone marrow from control and early-Abx mice (n=6 per group). Each symbol represents data from an individual mouse, and error bars represent SEM per group in one experiment. Data were analyzed using two-tailed Student's t-test. *, $P < 0.05$; **, $P < 0.01$; ***, $P < 0.001$; ****, $P < 0.0001$; ns, no significance. All experiments were repeated for two or three independent experiments. Source data are provided as a Source Data file.

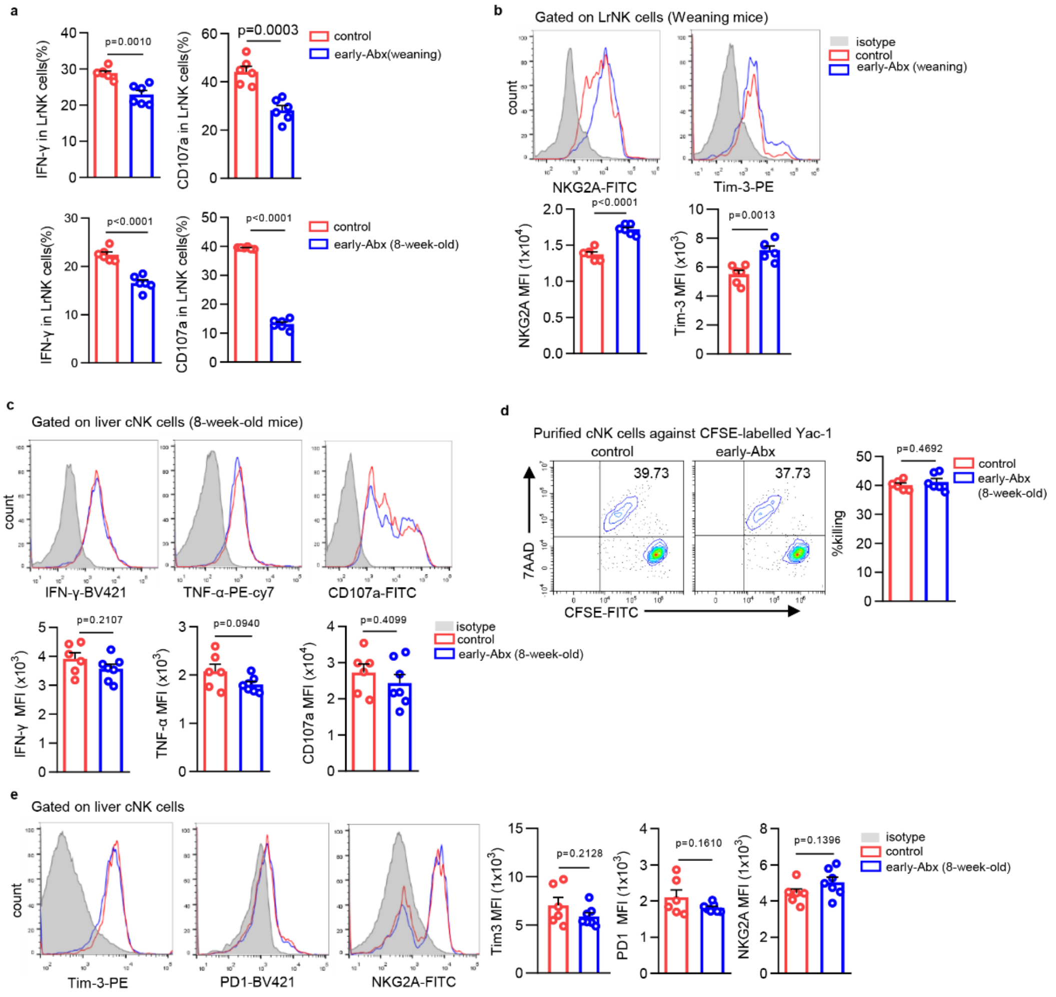

Supplementary Figure 3 The effect of early-Abx on liver cell function and phenotype.

(a) Bar graph and for the percentage of IFN-γ and CD107a in PMA and Ion-stimulated LrNK cells from weaning and 8-week-old control or early-Abx mice (n=6 per group). **(b)** Representative FACS plots and bar graph for the expression (MFI) of Tim-3 and NKG2A in LrNK cells from weaning control and early-Abx mice (n=6 per group). **(c)** Representative FACS plots bar graph and for the expression (MFI) of effector molecules in PMA and Ion-stimulated cNK cells from control and early-Abx mice (control n=6 and early-Abx n=7). **(d)** Cytotoxicity of cNK cells against CFSE-labeled YAC-1 cells. 7-AAD$^+$ CFSE$^+$ cells represented the killed cells (n=6 per group). **(e)**

Representative FACS plots and bar graph for the expression (MFI) of Tim-3, PD1 and NKG2A in cNK cells from control and early-Abx mice (control n=6 and early-Abx n=7). Each symbol represents data from an individual mouse, and error bars represent SEM per group in one experiment. Data were analyzed using two-tailed Student's t-test. **, $P < 0.01$; ***, $P < 0.001$; ****, $P < 0.0001$; ns, no significance. Source data are provided as a Source Data file.

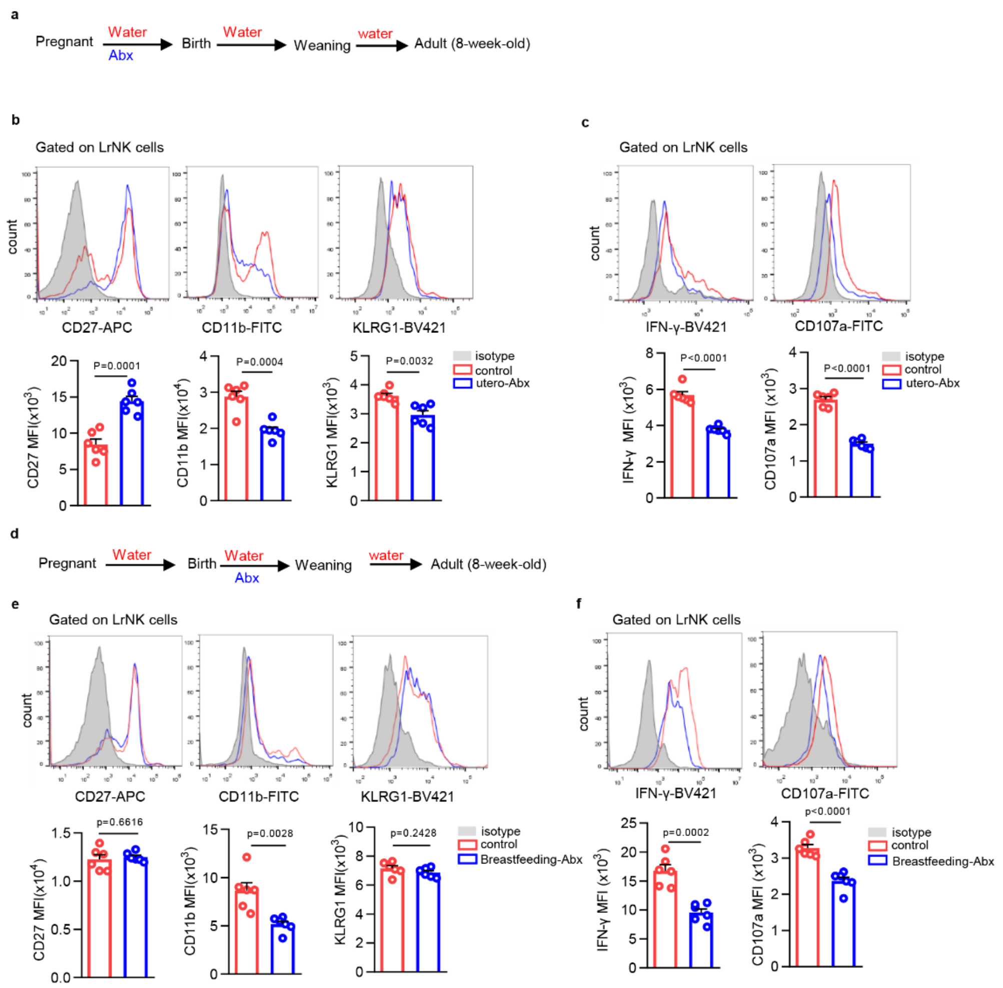

Supplementary Figure 4 Impaired LrNK cell maturation and function by Abx exposure during pregnancy or breastfeeding period.

(a) Schemes of utero-Abx mouse model experimental design. **(b)** Representative FACS plots and bar graph for the expression level (MFI) of CD27, CD11b and KLRG1 in LrNK cell subsets from control and utero-Abx mice (n=6 per group). **(c)** Representative FACS plots and bar graph for the expression (MFI) of IFN-γ and CD107a in PMA and Ion-stimulated LrNK cells from control and utero-Abx mice (n=6 per group). **(d)** Schemes of Breastfeeding-Abx mouse model experimental design. **(e)** Representative FACS plots and bar graph for the expression level (MFI) of CD27, CD11b and KLRG1 in LrNK cell subsets from control and Breastfeeding-Abx mice (n=6

per group). **(f)** Representative FACS plots and bar graph for the expression (MFI) of IFN-γ and CD107a in PMA and Ion-stimulated LrNK cells from control and Breastfeeding-Abx mice (n=6 per group). Each symbol represents data from an individual mouse, and error bars represent SEM per group in one experiment. Data were analyzed using two-tailed Student's t-test (two-tail unpaired t-test). **, $P < 0.01$; ***, $P < 0.001$; ****, $P < 0.0001$; ns, no significance. Source data are provided as a Source Data file.

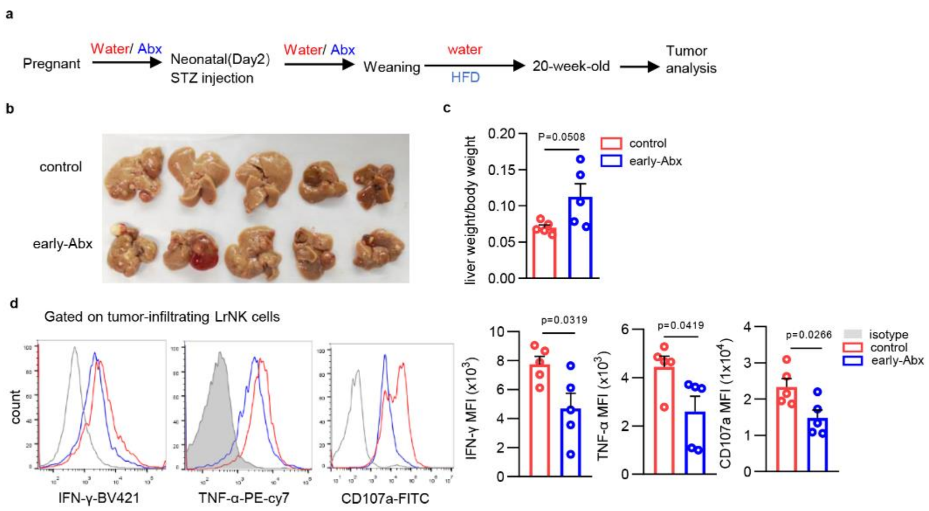

Supplementary Figure 5 Early-Abx exposure promotes STZ-HFD-induced HCC progression.

(a) Experimental scheme of STZ-HFD-induced HCC mouse model (n=5 per group). **(b-c)** *In vitro* tumor imaging (b) and liver /body weight (c) was shown (n=5 per group). **(d)** Representative FACS plots bar graph and for the expression (MFI) of effector molecules in PMA and Ion-stimulated LrNK cell subsets from control and early-Abx HCC mice (n=5 per group). Each symbol represents data from an individual mouse, and error bars represent SEM per group in one experiment. Data were analyzed using two-tailed Student's t-test. *, $P < 0.05$. All experiments were repeated for two to three times. Source data are provided as a Source Data file.

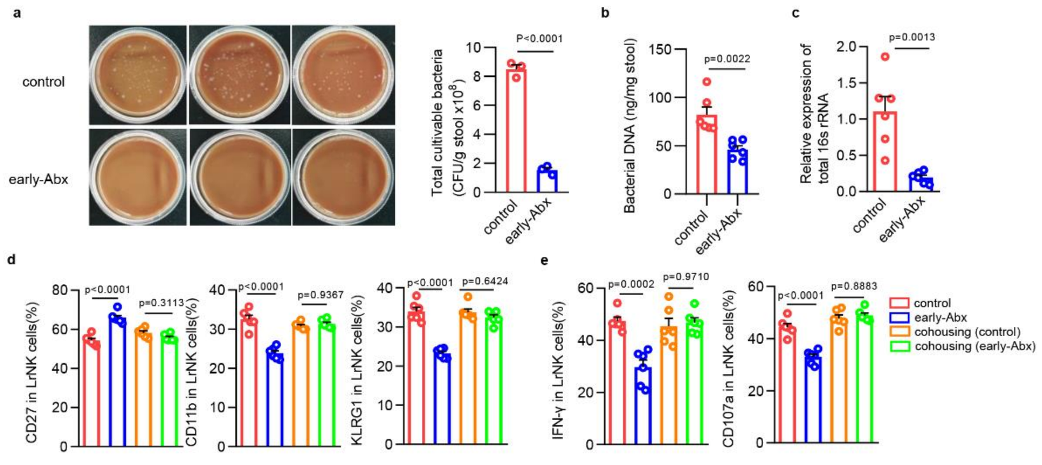

Supplementary Figure 6 Early-Abx treatment-induced dysbiosis dampens LrNK cell functional maturation.

(a) Photograph showing total cultivable bacteria of faeces taken from control and early-Abx mice on a blood agar plate in 10^{-5} dilution (n=3 per group). **(b)** Determination of fecal genomic bacterial DNA content (n=6 per group). **(c)**The total content of 16s rRNA in feces of control group and early Abx mice with the same quality was analyzed by qPCR (n=6 per group). **(d)** Bar graph for the percentage of CD27, CD11b and KLRG1 on LrNK cells from the four groups of mice (n=6 per group). **(e)** Bar graph for the percentage of IFN-γ and CD107a in PMA and Ion-stimulated LrNK cells from the four groups of mice (n=6 per group). Each symbol represents data from an individual mouse, and error bars represent SEM per group in one experiment. Data were analyzed using two-tailed Student's t-test (a-c) or one-way ANOVA with Tukey's multiple comparisons test (d-e). **, P < 0.01; ***, P < 0.001; ****, P < 0.0001, ns, no significance. Source data are provided as a Source Data file.

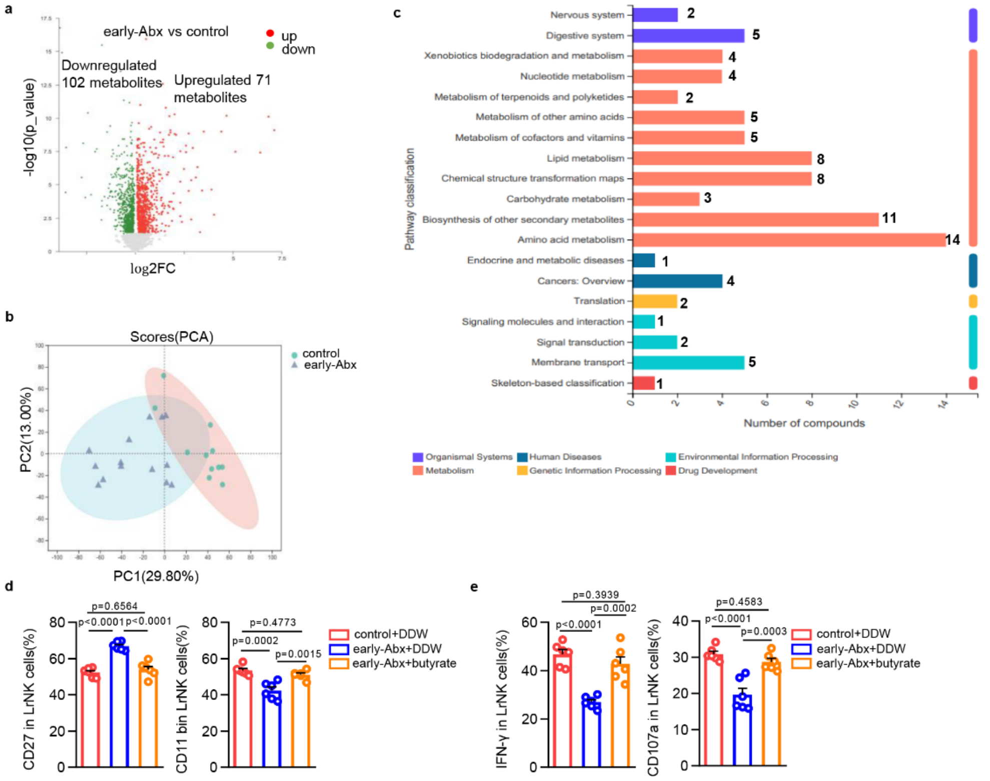

Supplementary Figure 7 Alterations of microbial metabolites are responsible for the impairment of LrNK cell maturation and function induced by early-Abx.

LC-MS analysis of microbiota metabolites from control and early-Abx mice (control n=11 early-Abx n=14). **(a)** Volcano plot showing the differences in faecal metabolites between control and earl-Abx mice. **(b)** PCoA ordination plot on weighted UniFrac distance matrix. Each subject is identified by a point. Control mice are represented in green (right), early-Abx mice are represented in blue(left). **(c)** KEGG pathway showing the enrichment of differentially expressed metabolites in multiple metabolic pathways between control and earl-Abx mice. **(d)** Bar graph for the percentage of CD27 and CD11b on LrNK cells from the different groups of mice (n=6 per group). **(e)** Bar graph for the percentage of IFN-γ and CD107a in PMA and Ion-stimulated LrNK cells from the different groups of mice (n=6 per group). Each symbol represents data from an

individual mouse, and error bars represent SEM per group in one experiment. Data were analyzed using two-tailed Student’s t-test (a) or one-way ANOVA with Tukey's multiple comparisons test (d-e). **, $P < 0.01$; *** $P < 0.001$; ****, $P < 0.0001$, ns, no significance. Source data are provided as a Source Data file.

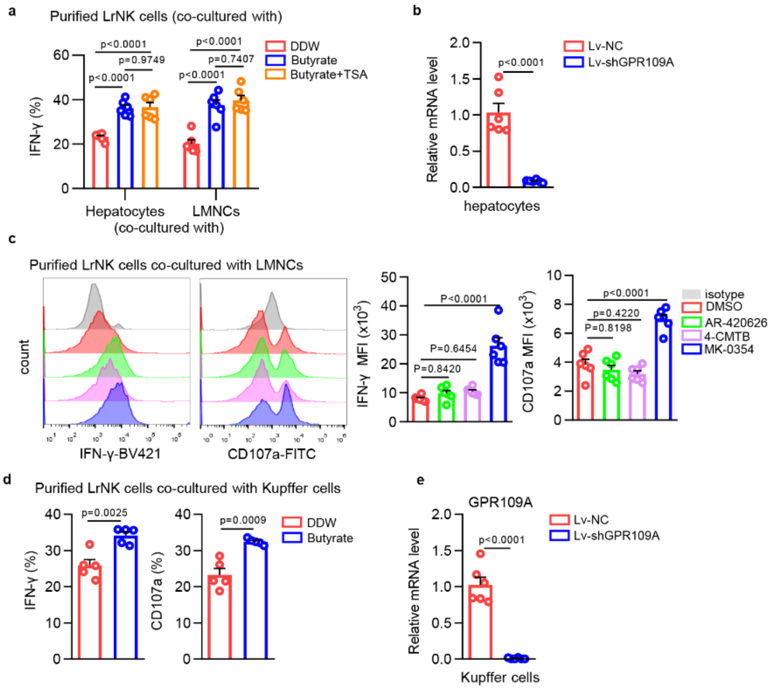

Supplementary Figure 8 Butyrate/GPR109A axis enhances LrNK cell functional maturation through regulating liver microenviroment.

(a) Purified LrNK cells were co-cultured with LMNCs or hepatocytes and treated with DDW, butyrate or butyrate plus TSA for 24h and then stimulated with poly(I:C) for 16h. Percentage of IFN-γ in LrNK cells were shown (n=6 per group).

(b) RT-qPCR analysis for GPR109A expression in control or GPR109A shRNA lentivirus (Lv-GPR109A) infected hepatocytes (n=6 per group). **(c)** Representative FACS plots and bar graph for the expression (MFI) of IFN-γ and CD107a in LrNK cells co-cultured with LMNCs in the presence of DMSO, AR-420626 (10uM), 4-CMTB (10uM) or MK-0354 (100uM) (n=6 per group). **(d)** Purified LrNK cells were co-cultured with Kupffer cells and treated with DDW or butyrate for 24h and then stimulated with poly(I:C) for 16h. The percentage of IFN-γ and CD107a in LrNK cells were shown (n=5 per group). **(e)** RT-qPCR analysis for GPR109A expression in control or GPR109A

shRNA lentivirus (Lv-GPR109A) infected Kupffer cells (n=6 per group). Each symbol represents data from an individual mouse, and error bars represent SEM per group in one experiment. Data were analyzed using two-tailed Student's t-test (a, c, e, f) or one-way ANOVA with Tukey's multiple comparisons test (b, d). **, P < 0.01; ***, P < 0.001; ****, P < 0.0001; ns, no significance. Source data are provided as a Source Data file.

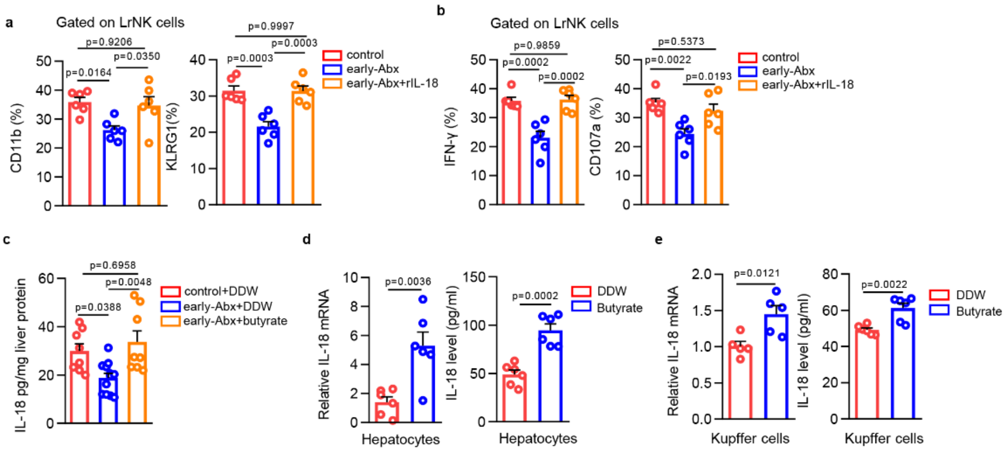

Supplementary Figure 9 IL-18 involves in the regulation of LrNK cell maturation and function by butyrate.

(a-b) The percentage of maturation markers (a) or effector molecules (b) in LrNK cells from control or early-Abx mice with or without recombinant IL-18 treatment (n=6 per group). **(c)** ELISA assay for IL-18 levels in liver tissues from different groups of mice (control n=8, early-Abx n=12, early-Abx + butyrate n=8). **(d-e)** RT-qPCR and ELISA assay for IL-18 expression in hepatocytes (d) or Kupffer cells (e) treated with DDW or butyrate for 24h (n=6 per group). Each symbol represents data from an individual mouse, and error bars represent SEM per group in one experiment. Data were analyzed using two-tailed Student's t-test (b, c, d, e) or one-way ANOVA with Tukey's multiple comparisons test (a). *, P < 0.05; **, P < 0.01; ***, P < 0.001; ns, no significance. All experiments were repeated for two to three times. Source data are provided as a Source Data file.

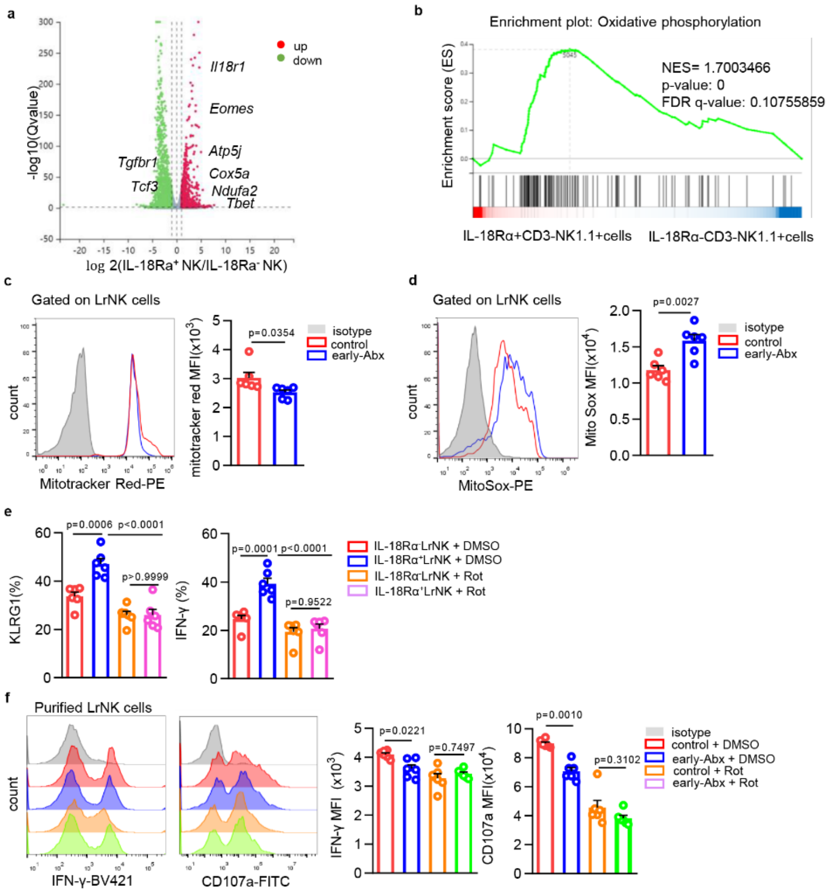

Supplementary Figure 10 IL-18 modulates LrNK cell maturation and function through enhancing oxidative phosphorylation.

(a-b) RNA-seq with purified IL-18Rα^-NK and IL-18Rα^+NK cells from WT mice. **(a)** Volcano plot (log 2(Fold Change) vs -log10(Q-Value)). Fold change was determinant as $Log2$ MEANFPKM (IL-18Rα^+ NK/ IL-18Rα^- NK). **(b)** GSEA of oxidative phosphorylation related genes. **(c-d)** Representative FACS plots and bar graph for the mitochondrial membrane potential, indicated by Mitotracker Red staining (c) and mitochondrial ROS levels measured by MitoSOX (d) in LrNK cells from control or early-Abx mice (n=6 per group). **(e)** Bar graph for the percentage of KLRG1 and IFN-γ in purified IL-18Rα^-LrNK or IL-18Rα^+LrNK cells treated with DMSO or Rotenone (n=6 per group). **(f)** Representative FACS plots and bar graph for the expression (MFI) of IFN-

γ and CD107a in LrNK cells purified from control or early-Abx mice and treated with DMSO or Rotenone (n=6 per group). Dots represent data from individual mice, and error bars represent SEM per group in one experiment. Data were analyzed using two-tailed Student's t test (a, c, d), Kolmogorov-Smirnov test (b) or one-way ANOVA with Tukey's multiple comparisons test (e, f). *, $P < 0.05$; **, $P < 0.01$; ***, $P < 0.001$; ****, $P < 0.0001$; ns, no significance. Source data are provided as a Source Data file.

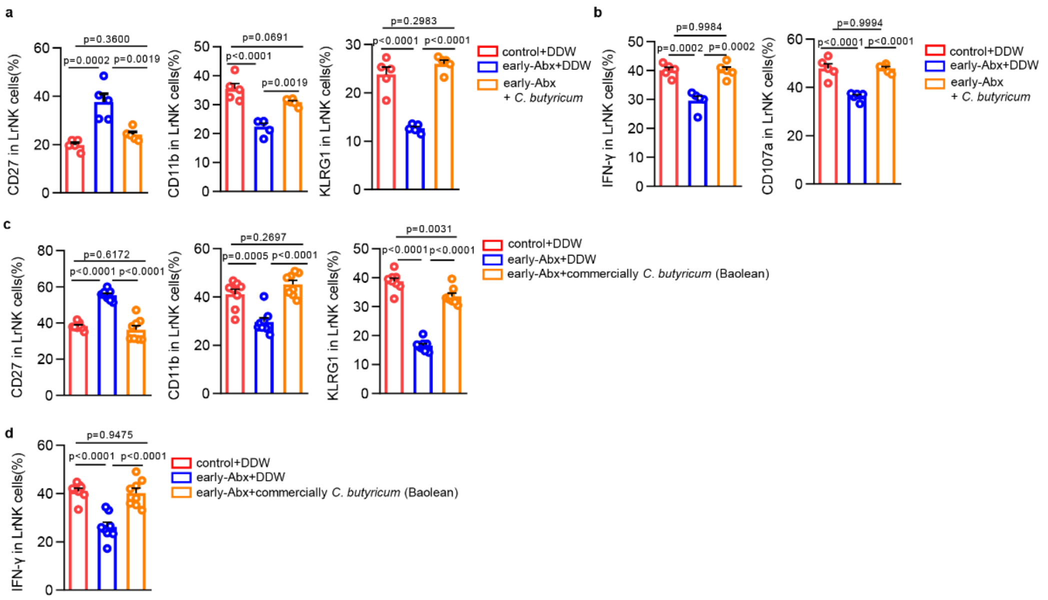

Supplementary Figure 11 Dietary Clostridium butyricum restores LrNK maturation and function in early-Abx treated mice.

(a-b) Percentage of the maturation markers CD27,CD11b and KLRG1 (a) and effector molecules IFN-γ andCD107a (b) in LrNK cells from different groups of mice (n=5 per group). **(c-d)** Percentage of the maturation markers CD27,CD11b and KLRG1 (c) and effector molecules IFN-γ (d) in LrNK cells from different groups of mice (n=8 per group). Dots represent data from individual mice, and error bars represent SEM per group in one experiment. Data were analyzed using one-way ANOVA with Tukey's multiple comparisons test (a-d). **, P < 0.01; ***, P < 0.001; ****, P < 0.0001; ns, no significance. Source data are provided as a Source Data file.

Supplementary Table 1. Antibodies used for flow cytometry

Antibodies	SOURCE	IDENTIFIER	Dilutions
PE anti-mouse CD3	Biolegend	Clone: 17A2	0.1μg/100μl
PEcy7 anti-mouse CD3	Biolegend	Clone:1452C11	0.1μg/100μl
BV421 anti-mouse CD3	Biolegend	Clone： 17A2	0.1μg/100μl
APC anti-mouse CD3	Biolegend	Clone： 17A2	0.1μg/100μl
APC anti-mouse NK1.1	BD	Clone: PK136	0.1μg/100μl
PEcy7 anti-mouse NK1.1	Biolegend	Clone： PK136	0.1μg/100μl
FITC anti-mouse NK1.1	Biolegend	Clone： S17016D	0.1μg/100μl
APC anti-mouse CD27	Biolegend	Clone: LG.3A10	0.1μg/100μl
PE anti-mouse CD122	Biolegend	Clone: 5H4	0.1μg/100μl
APC anti-mouse CD122	Biolegend	Clone: 5H4	0.1μg/100μl
FITC anti-mouse Lineage (CD3, Gr-1, CD11b, CD45R, Ter119)	Biolegend	Clone: 145-2C11	0.1μg/100μl
FITC anti-mouse/human CD11b	Biolegend	Clone： M1/70	0.1μg/100μl
PE anti-mouse CD45.1	Biolegend	Clone: A20	0.1μg/100μl
FITC anti-mouse CD45.1	Biolegend	Clone: A20	0.1μg/100μl
FITC anti-mouse CD45.2	Biolegend	Clone: 104	0.1μg/100μl
BV421 anti-mouse CD45.2	Biolegend	Clone: 104	0.1μg/100μl
FITC anti-mouse IFN-γ	Biolegend	Clone: XMG1.2	0.2μg/100μl
BV421 anti-mouse IFN-γ	Biolegend	Clone: XMG1.2	0.2μg/100μl
PE/cy7 anti-mouse TNF-α	Biolegend	Clone: MP6-XT22	0.2μg/100μl
BV421 anti-mouse CD107a	Biolegend	Clone: 1D4B	0.2μg/100μl
FITC anti-mouse CD107a	Biolegend	Clone: 1D4B	0.2μg/100μl
BV421 anti-mouse KLRG1	Biolegend	Clone: 2F1/KLRG1	0.2μg/100μl
BV421anti-mouse CD279/PD1	Biolegend	Clone: 29F.1A12	0.1μg/100μl
APC anti-mouse perforin	Biolegend	Clone: S16009A	0.2μg/100μl
FITC anti-mouse NKG2A/C/E	BD	Clone: 20d5	0.1μg/100μl
PE anti-mouse Tim3	Biolegend	Clone: B8.2C12	0.1μg/100μl
BV421 anti-mouse TIGIT(Vstm3)	Biolegend	Clone: 1G9	0.1μg/100μl
Alexa 647 anti-Mouse CD49a	BD	Clone： Ha31/8	0.2μg/100μl
PE anti-mouse CD49b	Biolegend	Clone： DX5	0.2μg/100μl
PE anti-mouse CD218a (IL-18Rα)	Biolegend	Clone： A17071D	0.2μg/100μl
PE anti-mouse CD200R	Biolegend	Clone： OX-110	0.1μg/100μl
MitoTracker™ Red	Invitrogen	Cat#: M7512	50nmol
MitoSOX™ Red	Invitrogen	Cat#: M36008	50nmol
PE anti-mouse EOMEs	eBioscience	Cat#: 00-5523-00	0.2μg/100μl
PE anti-mouse CD253/Trail	eBioscience	Cat#: 12-5951-81	0.2μg/100μl
PEcy7 anti-mouse CD69	eBioscience	Cat#: H1.2F3	0.1μg/100μl
BV421 anti-mouse Ly-6A/E(Sca-1)	Biolegend	Cat#: 108127	0.1μg/100μl
PECy7 anti-mouse CD244.2	Biolegend	Cat#: 133512	0.1μg/100μl
BV421 anti-mouse CD127	Biolegend	Cat#: 135023	0.1μg/100μl
PE anti-mouse CD135	Biolegend	Cat#: 135305	0.1μg/100μl

FITC anti-BrdU	Biolegend	Cat#: 364103	0.2μg/100μl
7AAD	Thermo fisher	Cat#: 00-6993-50	0.2μg/100μl
FITC AnnexinV	Biolegend	Cat#: 640906	5μl /100μl
BV510 anti-human CD45	Biolegend	Cat#: 304036	0.2μg/100μl
FITC anti-human CD3	Biolegend	Cat#: 300406	1μl /100μl
PE anti-human CD56	Biolegend	Cat#: 318306	1μl /100μl
Alexa647 anti-human CD49a	Biolegend	Cat#: 328310	1μl /100μl
percpcy5.5 anti-human CD27	Biolegend	Cat#: 124214	1μl /100μl
BV421 anti-human IFN-γ	Biolegend	Cat#: 506538	1μl /100μl
FITC anti-human CD107a	Biolegend	Cat#: 328606	1μl /100μl

Supplementary Table 2. Sequences of primers used for RT-qPCR.

Primer	Sequences
m-Il-18	F: GACTCTTGCGTCAACTTCAAGG
	R: CAGGCTGTCTTTTGTCAACGA
m-Hcar2	F: CTGGAGGTTCGGAGGCATC
	R: TCGCCATTTTTGGTCATCATGT
m-Ffar3	F: CTTCTTTCTTGGCAATTACTGGC
	R: CCGAAATGGTCAGGTTTAGCAA
m-Ffar2	F: CTTGATCCTCACGGCCTACAT
	R: CCAGGGTCAGATTAAGCAGGAG
m-Il2	F: TGAGCAGGATGGAGAATTACAGG
	R: GTCCAAGTTCATCTTCTAGGCAC
m-Il12	F: TGGTTTGCCATCGTTTTGCTG
	R: ACAGGTGAGGTTCACTGTTTCT
m-Il15	F: ACATCCATCTCGTGCTACTTGT
	R: GCCTCTGTTTTAGGGAGACCT
m- Ifng	F: GAGCTCATTGAATGCTTGGC
	R: GCGTCATTGAATCACACCTG
m-Ccl3	F: TTCTCTGTACCATGACACTCTGC
	R: CGTGGAATCTTCCGGCTGTAG
m-Ccl8	F: TCTACGCAGTGCTTCTTTGCC
	R: AAGGGGGATCTTCAGCTTTAGTA

m-Cxcl11	F: GGCTTCCTTATGTTCAAACAGGG<br>R: GCCGTTACTCGGGTAAATTACA
m-Ccl12	F: ATTTCCACACTTCTATGCCTCCT<br>R: ATTTCCACACTTCTATGCCTCCT
Total 16S	F: ACTCCTACGGGAGGCAGCAG<br>R: ATTACCGCGGCTGCTGG
m-Socs2	F: TGTCCAGATGTGCAAGGATAAA<br>R: ATGGCGAGTCGACAGAAATG
m-Zfp683	F: CTCAGCCACTTGCAGACTCA<br>R: CTGTCGGTGGAGGCTTTGTA
m-Rorα	F: CTCGCTAGAGGTGGTGTTTATT<br>R: TCGCATACTTCCCGTCAAAG
m-Ahr	F: AAGCAACACTAGCAGGAAAGA<br>R: GCTTGAAGGAGGACACAGATAG
m-Tbx21	F: GAGACACTAAGAGGAGGAGGAT<br>R: TGGCCTTCGGTTTCCTTATC
