# Supplementary material for: Early life gut microbiota sustains liver-resident natural killer cells maturation via the butyrate-IL-18 axis
Source: Nat Commun. 2023 Mar 27;14:1710. doi: 10.1038/s41467-023-37419-7 (PMC10043027; doi:10.1038/s41467-023-37419-7)
Supplement: Supplementary file 3 — Reporting Summary [file 41467_2023_37419_MOESM3_ESM.pdf]

nature portfolio

Corresponding author(s): Xiaohong Liang, Chunhong Ma

Last updated by author(s): Feb 28, 2022

Reporting Summary

Nature Portfolio wishes to improve the reproducibility of the work that we publish. This form provides structure for consistency and transparency in reporting. For further information on Nature Portfolio policies, see our Editorial Policies and the Editorial Policy Checklist.

Statistics

For all statistical analyses, confirm that the following items are present in the figure legend, table legend, main text, or Methods section.

n/a	Confirmed	
☐	☒	The exact sample size (*n*) for each experimental group/condition, given as a discrete number and unit of measurement
☐	☒	A statement on whether measurements were taken from distinct samples or whether the same sample was measured repeatedly
☐	☒	The statistical test(s) used AND whether they are one- or two-sided<br>*Only common tests should be described solely by name; describe more complex techniques in the Methods section.*
☒	☐	A description of all covariates tested
☐	☒	A description of any assumptions or corrections, such as tests of normality and adjustment for multiple comparisons
☐	☒	A full description of the statistical parameters including central tendency (e.g. means) or other basic estimates (e.g. regression coefficient) AND variation (e.g. standard deviation) or associated estimates of uncertainty (e.g. confidence intervals)
☐	☒	For null hypothesis testing, the test statistic (e.g. *F*, *t*, *r*) with confidence intervals, effect sizes, degrees of freedom and *P* value noted<br>*Give P values as exact values whenever suitable.*
☒	☐	For Bayesian analysis, information on the choice of priors and Markov chain Monte Carlo settings
☒	☐	For hierarchical and complex designs, identification of the appropriate level for tests and full reporting of outcomes
☒	☐	Estimates of effect sizes (e.g. Cohen's *d*, Pearson's *r*), indicating how they were calculated

Our web collection on statistics for biologists contains articles on many of the points above.

Software and code

Policy information about availability of computer code

Data collection	Flow cytometry data were acquired with beckman CytoFLEX S.<br>qRT-PCR data were acquired with biorad CFX-Connect<br>The oxygen consumption rate (OCR) data were acquired with XF96 Seahorse Extracellular Flux Analyzer (Agilent).<br>16s rRNA seq were acquired on the Illumina Hiseq platform with Majorbio
Data analysis	Flow cytometry data were analysed with FlowJo 10.6.2 or CytExpert 2.3.0.<br>The oxygen consumption rate (OCR) data were analysed with Seahorse Bioscience Wave.<br>16s rRNA data were analyzed on the online platform of Majorbio Cloud Platform (www.majorbio.com).<br>Statistics: Graphpad Prism 8.

For manuscripts utilizing custom algorithms or software that are central to the research but not yet described in published literature, software must be made available to editors and reviewers. We strongly encourage code deposition in a community repository (e.g. GitHub). See the Nature Portfolio guidelines for submitting code & software for further information.

Data

Policy information about availability of data

All manuscripts must include a data availability statement. This statement should provide the following information, where applicable:

- Accession codes, unique identifiers, or web links for publicly available datasets
- A description of any restrictions on data availability
- For clinical datasets or third party data, please ensure that the statement adheres to our policy

Source data are provided with this paper. The 16S rRNA sequencing datasets were uploaded to the NCBI database with SRA, accession:PRJNA890468. RNA-seq data have been deposited in NCBI database under the accession number PRJNA884315. The raw data are provided as a Source Data file. The authors declare that all data supporting the findings of this study are available within the paper and its Supplementary Information files or from the corresponding author upon reasonable request.

Human research participants

Policy information about studies involving human research participants and Sex and Gender in Research.

Reporting on sex and gender	Human normal liver tissue included in this study were obtained from 10 (5 females and 5 males) hepatic hemangioma patients in Qilu hospital, Shandong University and Shandong Provincial Hospital, and all patients provided informed written consent. Subjects elegible for this study were of all sexes (aged 26-65).
Population characteristics	The normal liver tissues used in this study were from diagnosed hepatic hemangioma patients (aged 26-65).
Recruitment	Human liver tissue included in this study were obtained from 10 hepatic hemangioma patients in Qilu hospital, Shandong University and Shandong Provincial Hospital from May 2022 to September 2022. The authors do not see any potential bias in the generation or interpretation of the data reported in this study.
Ethics oversight	All human tissues used in this study were approved by the Ethics Committee of Shandong University Schoo of Basic Medical Sciences.

Note that full information on the approval of the study protocol must also be provided in the manuscript.

Field-specific reporting

Please select the one below that is the best fit for your research. If you are not sure, read the appropriate sections before making your selection.

☒ Life sciences ☐ Behavioural & social sciences ☐ Ecological, evolutionary & environmental sciences

For a reference copy of the document with all sections, see nature.com/documents/nr-reporting-summary-flat.pdf

Life sciences study design

All studies must disclose on these points even when the disclosure is negative.

Sample size	Sample sizes were chosen based on preliminary data demonstrating statistically significant differences for each specific assay
Data exclusions	No data was excluded.
Replication	For animal experiments, at least 5 mice were included for each group, and independent experimental repeats were performed two or three times to ensure reproducibility of the results. For in vitro experiment, replicates were not less than three in each group. All the replications showed the similar results.
Randomization	For mouse experiment, mice were mated after 8 weeks of age and 4~5 male littermates from at least 2 dams were randomly assigned to different cages at post-weaning. Meanwhile, the offspring mice at same age and same gender from pregnant C57BL/6 dams without antibiotic treatment were maintained in parallel as controls in each experiment.<br>For cell experiment in vitro, cells were randomly allocated to the experimental groups.
Blinding	We had no specific methods to blind the investigators during the experiments, but all mice were treated equally at the same time

Reporting for specific materials, systems and methods

We require information from authors about some types of materials, experimental systems and methods used in many studies. Here, indicate whether each material, system or method listed is relevant to your study. If you are not sure if a list item applies to your research, read the appropriate section before selecting a response.

Materials & experimental systems

n/a	Involved in the study
☐	☒ Antibodies
☐	☒ Eukaryotic cell lines
☒	☐ Palaeontology and archaeology
☐	☒ Animals and other organisms
☒	☐ Clinical data
☒	☐ Dual use research of concern

Methods

n/a	Involved in the study
☒	☐ ChIP-seq
☐	☒ Flow cytometry
☒	☐ MRI-based neuroimaging

Antibodies

Antibodies used

PE anti-mouse CD3 Biolegend Clone: 17A2
PEcy7 anti-mouse CD3 Biolegend Clone:1452C11
BV421 anti-mouse CD3 Biolegend Clone：17A2
APC anti-mouse CD3 Biolegend Clone：17A2
APC anti-mouse NK1.1 BD Clone: PK136
PEcy7 anti-mouse NK1.1 Biolegend Clone：PK136
FITC anti-mouse NK1.1 Biolegend Clone：S17016D
APC anti-mouse CD27 Biolegend Clone: LG.3A10
PE anti-mouse CD122 Biolegend Clone: 5H4
APC anti-mouse CD122 Biolegend Clone: 5H4
FITC anti-mouse Lineage (CD3, Gr-1, CD11b, CD45R, Ter119) Biolegend Clone: 145-2C11
FITC anti-mouse/human CD11b Biolegend Clone：M1/70
PE anti-mouse CD45.1 Biolegend Clone: A20
FITC anti-mouse CD45.1 Biolegend Clone: A20
FITC anti-mouse CD45.2 Biolegend Clone: 104
BV421 anti-mouse CD45.2 Biolegend Clone: 104
FITC anti-mouse IFN-γ Biolegend Clone: XMG1.2
BV421 anti-mouse IFN-γ Biolegend Clone: XMG1.2
PE/cy7 anti-mouse TNF-α Biolegend Clone: MP6-XT22
BV421 anti-mouse CD107a Biolegend Clone: 1D4B
FITC anti-mouse CD107a Biolegend Clone: 1D4B
BV421 anti-mouse KLRG1 Biolegend Clone: 2F1/KLRG1
BV421anti-mouse CD279/PD1 Biolegend Clone: 29F.1A12
APC anti-mouse perforin Biolegend Clone: S16009A
FITC anti-mouse NKG2A/C/E BD Clone: 20d5
PE anti-mouse Tim3 Biolegend Clone: B8.2C12
BV421 anti-mouse TIGIT(Vstm3) BD Clone: 1G9
Alexa 647 anti-Mouse CD49a Biolegend Clone：Ha31/8
PE anti-mouse CD49b Biolegend Clone：DX5
PE anti-mouse CD218a (IL-18Rα) Biolegend Clone：A17071D
PE anti-mouse CD200R Biolegend Clone： OX-110
MitoTracker™ Red Invitrogen Cat#: M7512
MitoSOX™ Red Invitrogen Cat#: M36008
PE anti-mouse EOMEse Bioscience Cat#: 00-5523-00
PE anti-mouse CD253/Trail eBioscience Cat#: 12-5951-81
PEcy7 anti-mouse CD69 eBioscience Cat#: H1.2F3
BV421 anti-mouse Ly-6A/E(Sca-1) Biolegend Cat#: 108127
PECy7 anti-mouse CD244.2 Biolegend Cat#: 133512
BV421 anti-mouse CD127 Biolegend Cat#: 135023
PE anti-mouse CD135 Biolegend Cat#: 135305
FITC anti-BrdU Biolegend Cat#: 364103
7AAD Thermo fisher Cat#: 00-6993-50
FITC AnnexinV Biolegend Cat#: 640906
BV510 anti-human CD45 Biolegend Cat#: 304036
FITC anti-human CD3 Biolegend Cat#: 300406
PE anti-human CD56 Biolegend Cat#: 318306
Alexa647 anti-human CD49a Biolegend Cat#: 328310
percpcy5.5 anti-human CD27 Biolegend Cat#: 124214
BV421 anti-human IFN-γ Biolegend Cat#: 506538
FITC anti-human CD107a Biolegend Cat#: 328606

Validation	All antibodies' validation statements for the species and application can be found on the manufacturer's website.

Eukaryotic cell lines

Policy information about cell lines and Sex and Gender in Research

Cell line source(s)	The mouse cell line Yac-1 cells was donated by Shandong Academy of Medical Sciences.
Authentication	Yac-1 cells were used in vitro flow-based killing assay.
Mycoplasma contamination	All cells were tested negative for mycoplasma contamination before use.
Commonly misidentified lines (See ICLAC register)	No commonly misidentified cell lines were used in the study.

Animals and other research organisms

Policy information about studies involving animals; ARRIVE guidelines recommended for reporting animal research, and Sex and Gender in Research

Laboratory animals	C57BL/6 mice (6-8 weeks of age) were purchased from Beijing Vital River Laboratory Animal Technology. IL-18Rα knockout mice were gifted by prof. Wei Wang from Sichuan University. CD45.1 mice were kindly provided by Dr. Xiaolong Liu (Center for Excellence in Molecular Cell Science, CAS). All mice were maintained under specific pathogen-free conditions, and experiments were carried out under the Shandong University Laboratory Animal Center's approval. All mice were maintained under specific pathogen-free conditions with a 12-h light, 12-h dark cycle and given free access to food and water.<br>We analysed the function and maturation of LrNK cells both in weaning and 8-week-old control or early-Abx male mice. 8-week-old control or early-Abx male mice (18~22g body weight) were subjected to HCC models. IL-18Rα knockout mice and CD45.1 mice (8-week-old) were used in the experimental.
Wild animals	The study did not involve wild animals.
Reporting on sex	There is no gender difference for the effect of early-life micorbiota on LrNK functional maturation. We have added this information in Material and Methods section
Field-collected samples	The study did not involve field-collected samples.
Ethics oversight	Animal protocols were approved by the Shandong University Laboratory Animal Center. Animal Ethics Number: ECSBMSSDU2020-2-86.

Note that full information on the approval of the study protocol must also be provided in the manuscript.

Flow Cytometry

Plots

Confirm that:

- [x] The axis labels state the marker and fluorochrome used (e.g. CD4-FITC).
- [x] The axis scales are clearly visible. Include numbers along axes only for bottom left plot of group (a 'group' is an analysis of identical markers).
- [x] All plots are contour plots with outliers or pseudocolor plots.
- [x] A numerical value for number of cells or percentage (with statistics) is provided.

Methodology

Sample preparation	For isolation of liver mononuclear cells (LMNCs), the mouse livers were washed and passed through a 200-gauge stainless steel mesh. The single cells were washed, red blood cells were lysed and then the cell suspension were centrifuged over 40% Percoll gradient medium. For bone marrow mononuclear cells, bone marrow was obtained by flushing femurs and tibias and then RBC lysed and washed with PBS.<br>For cell surface staining, cell suspensions were incubated with the specific labeled antibodies for 30 min at 4°C. For intracellular staining, freshly isolated cells were stimulated with PMA (50ng/ml) (Sigma) and Ion (1μg/ml) (Biolegend) for 2h, or IL-12 (20ng/ml) (Proteintech) and IL-15 (50ng/ml) (Proteintech) for 16h, then cultured with Brefeldin A (BFA) (Biolegend) at a final concentration of 10 μg/mL for 4h. After surface staining, cells were fixed with intracellular fixation buffer for 20min, then permeabilized with permeabilization buffer for 10min. Intracellular staining was performed with antibodies diluted into permeabilization buffer. For CD107a staining, cells were incubated with CD107a antibody for 4h.<br>For purification of LrNK cells, Kupffer cells, IL-18Rα- LrNK and IL-18Rα+LrNK cells, LMNCs isolated as described above were stained with surface marker antibodies and subjected to Moflo Astrios EQ.
Instrument	Beckma CytoFLEX S, Moflo Astrios EQ

Software	CytExpert 2.3.0, FlowJo 10.6.2
Cell population abundance	For analysis by Beckma CytoFLEX S, cells were run to achieve >30000 events in the gated cell population.<br>For purification of LrNK cells, Kupffer cells, IL-18Rα- LrNK and IL-18Rα+LrNK cells by Moflo Astrios EQ, more than 95% cells are positive for the sorted markers, as verified by running flow after sorting to confirm the sorting efficiency.
Gating strategy	Gating was performed based on identifying a distinct population in FSC vs SSC plot. The gating strategy is provided in supplementary figure 1

☒ Tick this box to confirm that a figure exemplifying the gating strategy is provided in the Supplementary Information.
